# Induction and decay of functional complement-fixing antibodies by the RTS,S malaria vaccine in children, and a negative impact of malaria exposure

**DOI:** 10.1186/s12916-019-1277-x

**Published:** 2019-02-25

**Authors:** Liriye Kurtovic, Paul A. Agius, Gaoqian Feng, Damien R. Drew, Itziar Ubillos, Jahit Sacarlal, John J. Aponte, Freya J. I. Fowkes, Carlota Dobaño, James G. Beeson

**Affiliations:** 1 Burnet Institute, Melbourne, Australia; 2 Department of Immunology and Pathology, Monash University, Melbourne, Australia; 3 Department of Epidemiology and Preventative Medicine, Monash University, Melbourne, Australia; 4 ISGlobal, Hospital Clínic Universitat de Barcelona, Barcelona, Catalonia Spain; 5 Centro de Investigação em Saúde de Manhiça, Maputo, Mozambique; 6 Faculdade de Medicina, Universidade Eduardo Mondlane (UEM), Maputo, Mozambique; 7 Centre for Epidemiology and Biostatistics, Melbourne School of Population and Global Health, The University of Melbourne, Melbourne, Australia; 8 Department of Microbiology, Monash University, Clayton, Australia; 9 Department of Medicine, The University of Melbourne, Parkville, Australia

**Keywords:** Antibody function, Circumsporozoite protein, Complement, Malaria, *Plasmodium falciparum*, RTS,S, Vaccines

## Abstract

**Background:**

Leading malaria vaccine, RTS,S, is based on the circumsporozoite protein (CSP) of sporozoites. RTS,S confers partial protection against malaria in children, but efficacy wanes relatively quickly after primary immunization. Vaccine efficacy has some association with anti-CSP IgG; however, it is unclear how these antibodies function, and how functional antibodies are induced and maintained over time. Recent studies identified antibody-complement interactions as a potentially important immune mechanism against sporozoites. Here, we investigated whether RTS,S vaccine-induced antibodies could function by interacting with complement.

**Methods:**

Serum samples were selected from children in a phase IIb trial of RTS,S/AS02_A_ conducted at two study sites of high and low malaria transmission intensity in Manhiça, Mozambique. Samples following primary immunization and 5-year post-immunization follow-up time points were included. Vaccine-induced antibodies were characterized by isotype, subclass, and epitope specificity, and tested for the ability to fix and activate complement. We additionally developed statistical methods to model the decay and determinants of functional antibodies after vaccination.

**Results:**

RTS,S vaccination induced anti-CSP antibodies that were mostly IgG1, with some IgG3, IgG2, and IgM. Complement-fixing antibodies were effectively induced by vaccination, and targeted the central repeat and C-terminal regions of CSP. Higher levels of complement-fixing antibodies were associated with IgG that equally recognized both the central repeat and C-terminal regions of CSP. Older age and higher malaria exposure were significantly associated with a poorer induction of functional antibodies. There was a marked decay in functional complement-fixing antibodies within months after vaccination, as well as decays in IgG subclasses and IgM. Statistical modeling suggested the decay in complement-fixing antibodies was mostly attributed to the waning of anti-CSP IgG1, and to a lesser extent IgG3.

**Conclusions:**

We demonstrate for the first time that RTS,S can induce complement-fixing antibodies in young malaria-exposed children. The short-lived nature of functional responses mirrors the declining vaccine efficacy of RTS,S over time. The negative influence of age and malaria exposure on functional antibodies has implications for understanding vaccine efficacy in different settings. These findings provide insights into the mechanisms and longevity of vaccine-induced immunity that will help inform the future development of highly efficacious and long-lasting malaria vaccines.

**Electronic supplementary material:**

The online version of this article (10.1186/s12916-019-1277-x) contains supplementary material, which is available to authorized users.

## Background

There is an unmet need for highly efficacious malaria vaccines, which would substantially reduce worldwide morbidity and mortality and accelerate malaria elimination. To date, only one candidate has been tested in phase III clinical trials, the RTS,S subunit vaccine administered with AS01_B_ adjuvant (liposome-based adjuvant). Thousands of children and infants were enrolled from multiple study sites across sub-Saharan Africa, and RTS,S was moderately efficacious against clinical malaria at 38–48 months follow-up (36.3% and 25.9% after the fourth booster at 20 months in children and infants, respectively) [1]. RTS,S has received a positive scientific opinion by the European Medicines Agency [2] and will undergo pilot implementation in areas of Ghana, Kenya, and Malawi to further evaluate vaccine safety, reduction in childhood mortality, and feasibility of the four-dose vaccine regimen [3, 4]. Despite this achievement, vaccine efficacy against clinical malaria was well below the target of 75% as set by the World Health Organization [5], and longitudinal studies show that protection rapidly declines after vaccination [1].

RTS,S is based on the major surface expressed antigen on *Plasmodium falciparum* sporozoites, the circumsporozoite protein (CSP). The vaccine construct is a fusion protein between a truncated form of CSP (containing the central repeat and C-terminal regions only) and hepatitis B surface antigen that is co-expressed with unfused hepatitis B surface antigen, which self-assemble as virus-like particles. The central repeat region is a tandem repeat of amino acid sequence NANP that is a known B cell epitope, and the C-terminal region contains B and T cell epitopes [6]. High levels of immunoglobulin G (IgG) to the central repeat region have been broadly associated with protection against clinical malaria in RTS,S vaccine trials [7]. A recent study was the first to report the specific IgG subclass responses induced by RTS,S/AS01_B_ in infants and young children (age groups, 6–12 weeks and 5–17 months) and identified that IgG3 to the central repeat region and IgG1 and IgG3 to the C-terminal region were associated with protection [8]. However, the mechanisms of such antibodies and how they confer protection are undefined. Further research is needed to understand the mechanisms of RTS,S-induced immunity and why efficacy is short-lived so that strategies to enhance vaccine efficacy and longevity in target populations can be developed.

Healthy malaria-naïve adults vaccinated with RTS,S develop antibodies that can block sporozoite infectivity and mediate opsonic phagocytosis using the THP-1 monocyte cell line in vitro. How this relates to protection is somewhat unclear, particularly for the latter mechanism, which has had conflicting findings [9–11]*.* Complement fixation and activation by antibodies is an important mechanism of humoral immunity to some viral and bacterial pathogens [12–14] and has been proposed to play a role in immunity against blood-stage *P. falciparum* infection [15]. We have recently demonstrated that naturally acquired human antibodies, or antibodies generated by repeated experimental inoculation of healthy volunteers with sporozoites, can promote fixation of complement factor C1q and activate the classical complement pathway against *P. falciparum* sporozoites [16, 17]. This antibody-complement interaction inhibited the traversal mode of sporozoite motility and led to cell death. Antibodies targeting CSP could also promote complement fixation [17]. Additionally, high levels of naturally acquired complement-fixing antibodies were associated with a reduced risk of clinical malaria in a longitudinal study of children [17]. This indicated that antibody-complement activity is an important mechanism of human immunity against sporozoites and raised the question of whether it may also be a functional mechanism induced by RTS,S.

Here, we examined antibody responses from a phase IIb clinical trial of RTS,S administered with AS02_A_ adjuvant (formerly used adjuvant that is an oil-in-water emulsion) in children resident in Manhiça, Mozambique, that was conducted at two study sites of different malaria transmission intensity [18]. We aimed to characterize RTS,S-induced antibodies by isotype, IgG subclass, and reactivity to different CSP regions, and to determine whether antibodies induced by RTS,S function by fixing complement. Furthermore, we aimed to understand the influence of antibody types and levels of malaria exposure on complement-fixing activity. We then developed statistical models of the decay of complement-fixing antibodies, IgG subclass responses, and IgM over 5 years of follow-up and explored which responses influenced the decay of complement-fixing antibodies.

## Methods

### Study participants

Serum samples were obtained from a previously conducted randomized control phase IIb clinical trial of the RTS,S/AS02_A_ vaccine (ClinicalTrials.gov registry number NCT00197041). The study was conducted in Mozambique at two study sites to evaluate protective efficacy against clinical disease (Manhiça, cohort 1) and new infection (Ilha Josina, cohort 2). Notably, malaria transmission intensity was low to moderate at the Manhiça study site and high at the Ilha Josina study site. Children aged 1 to 4 years were enrolled and administered three doses of RTS,S/AS02_A_ at months 0, 1, and 2 of the study or comparator vaccines as previously described [18]. We tested sera from a random selection of participants collected at baseline (month 0, M0) and 30 days after receiving the third and final RTS,S vaccination (month 3, M3) from Manhiça (RTS,S *N* = 50, comparator *N* = 25) and Ilha Josina (RTS,S *N* = 49, comparator *N* = 24) cohorts. We additionally tested sera from the Manhiça cohort collected during a 5-year follow-up to assess long-lived immune responses (months 8.5, 21, 33, 45, and 63, *n* = 30). Sample selection was at random and summary statistics closely matched that of the entire cohort (median age, 2.8 years and 2.5 years; male-to-female ratio, 0.93 and 0.83; Manhiça malaria negative-to-positive ratio, 0.17 and 0.15; Ilha Josina malaria negative-to-positive ratio, 2.32 and 1.41, respectively). Individuals tested at later time points were randomly selected from those with sufficient volumes at all time points (out of *n* = 371). Note that all serum samples were heat treated for 45 min at 56 °C to inactivate any host complement proteins.

### Experimenter blinding

Experimenters were blinded from the vaccine group, age group, and study site until all participants had been tested for IgG, immunoglobulin M (IgM), and C1q-fixation to CSP.

### Antigens

The following antigens used in this study were all based on *P. falciparum* 3D7: recombinant CSP beginning at amino acid residue 50 that contained (NANP)_22_ repeats and (NVPD)_4_ repeats expressed in *Pichia pastoris* (Sanaria, Rockville, USA), synthetic (NANP)_15_ peptide (NANP) representing the central repeat region of CSP (Life Tein, Hillsborough, USA), recombinant C-terminal region (CT) of CSP expressed in HEK293 cells at Burnet Institute (as described in Additional file 1: Figure S1), recombinant merozoite surface protein 2 (MSP2), and apical membrane antigen 1 (AMA1) both expressed in *Escherichia coli* as previously described [19, 20].

### Antibody detection assay

Sera were tested by standard enzyme-linked immunosorbent assay (ELISA) to detect antibody reactivity as follows. Ninety-six-well flat bottom MaxiSorp plates (Thermo Fisher Scientific, Waltham, USA) were coated in 0.5 μg/ml antigen in phosphate-buffered saline (PBS) overnight at 4 °C. Plates were washed (thrice with PBS-Tween20 0.05%) using the ELx405 automated plate washer (BioTeck, Winooski, USA) and blocked with 1% casein in PBS (*w*/*v*) for 2 h at 37 °C. Plates were washed and then incubated with human sera (tested in duplicate) in buffer (0.1% casein in PBS, *v*/*v*) for 2 h at room temperature (RT). Sera were tested at 1/4000 dilution for IgG and IgG subclass detection and 1/500 for IgM detection (note that for the decay analysis, samples collected between M3 and M63 were tested at 1/500 for IgG subclass detection). Plates were washed, and antibody isotypes were detected using goat anti-human IgG and IgM conjugated to horseradish peroxidase (HRP, Millipore, Burlington, USA) at 1/2500 dilution in buffer for 1 h at RT. Plates were washed for a final time and incubated with 2,2′-azino-bis(3-ethylbenzothiazoline-6-sulfonic acid) substrate (ABTS, Thermo Fisher Scientific) shielded from light at RT. IgG and IgM reactivities were measured after 20 and 60 min, respectively, at an optical density (OD) of 405 nm using the Multiskan Go plate reader (Thermo Fisher Scientific).

To detect IgG subclasses, plates were instead incubated with mouse anti-human IgG1, IgG2, IgG3, and IgG4 (Thermo Fisher Scientific), washed, and then incubated with goat anti-mouse IgG HRP (Millipore) all at 1/1000 dilution in buffer for 1 h at RT. Plates were washed for a final time and incubated with tetramethylbenzidine substrate (TMB, Thermo Fisher Scientific) shielded from light at RT. IgG subclass reactivity was stopped after 30 min of incubation using 1 M sulfuric acid, and OD was measured at 450 nm. We have previously confirmed the specificity and sensitivity of the IgG subclass reagents used [21]. A prior report of IgG subclass responses induced by RTS,S in the phase III trial [8] used a suspension bead array method rather than ELISA. While there may be differences between these methods, prior evaluation of the two approaches for detection of malaria antibodies using a range of antigens suggested the two methods were comparable [22].

### Complement fixation assay

Sera were tested for the ability to fix complement using the plate-based complement fixation assay as previously described [17]. Briefly, coating and blocking were conducted as described above for antibody detection. Plates were incubated with human sera (tested in duplicate) at 1/250 dilution in buffer for 2 h at RT (note that for the decay analysis, samples collected M3-M63 were re-tested at 1/110 dilution for C1q-fixation). Plates were washed and incubated with purified human C1q (Millipore) at 10 μg/ml in buffer for 30 min, followed by washing. To detect C1q-fixation, plates were incubated with rabbit anti-C1q IgG (in-house), washed, and then incubated with goat anti-rabbit IgG HRP (Millipore) both at 1/2000 dilution in buffer for 1 h at RT. Detection antibodies were validated as described in Additional file 1: Figure S2. Plates were washed for a final time and incubated with TMB shielded from light at RT. C1q-fixation reactivity was stopped after 30 min of incubation using 1 M sulfuric acid, and OD was measured at 450 nm.

We additionally tested a random selection of *n* = 20 individuals (from the RTS,S vaccine group collected at 3 M) for C5b-C9-fixation to CSP. This was conducted using the same protocol, but fresh human serum pooled from malaria-naïve donors was used as a source of complement (1/10 dilution), and rabbit anti-C5b-C9 detection antibodies were used (1/1000 dilution).

### Experimental controls and standardization

To ensure accurate results, human serum samples were tested in duplicate, and those with high variability (> 25%) were re-tested or excluded (unless duplicates differed OD < 0.1). Raw data were corrected for background reactivity using no-serum/blank negative controls and adjusted for plate-to-plate variation using malaria-exposed positive controls that were included on each plate. We also included malaria-naïve negative controls from Melbourne donors (*N* = 24), and test samples with an OD greater than the mean + 3 standard deviations of the Melbourne controls were considered positive.

We also examined the epitope specificity of individuals and considered the ratio of NANP-to-CT IgG as equal if the variability between NANP and CT responses was < 25%, which was the acceptable range for duplicate variability. Individuals who exceeded this were considered as having a NANP (ratio > 1.25) or CT-skewed (ratio < 0.75) IgG response.

### Statistical analysis

For descriptive and comparative analysis, the median and interquartile range (IQR) were reported, and the following two-tailed non-parametric tests were performed where appropriate (GraphPad Prism 7; *p* < 0.05 was considered significant): Mann-Whitney *U* test, Wilcoxon matched-pairs signed-rank test, and Spearman’s correlation coefficient (Rho).

Statistical models were developed to investigate (i) decay rates of complement-fixing antibodies, antibody isotypes, and IgG subclasses and (ii) the relationship between complement-fixing antibodies and different antibody types using repeated measures longitudinal data (Stata version 14, StataCorp). Given the dependency on the data from repeated measurement, multilevel modelling was used to estimate latent growth curve models exploring the subject-specific nature of the association between time and C1q-fixation, IgG subclasses, and IgM. Given the non-linear functional form of the association between time and C1q-fixation, IgG subclasses, and IgM, latent growth curve models regressed the log of C1q-fixation and IgG subclass and IgM on log time, and post-estimation non-linear equations using exponentiated model coefficients were used to provide proportional rates of change at specific time points. Latent growth curve models comprised two levels, individuals at level 2 (i.e., random intercept and coefficient for time) and their C1q-fixation, IgG subclass, and IgM response across time at level 1 (see Additional file 1: Supplementary equation 1). Nested model-based likelihood ratio statistics were used to provide statistical inference for model fit when relaxing model constraints (random effects and the functional form of fixed effects for time) and Akaike Information Criterion (AIC) and Bayesian Information Criterion (BIC) fit statistics used to inform functional form modeling across non-nested models. To further assess the fit of the estimated latent growth curve models, diagnostic plots comparing participants observed marker levels with Bayesian model-based (best linear unbiased predictions) predicted levels over time were produced and examined.

Contemporaneous (i.e., both outcome and factor variable responses from the same time period were regressed) unadjusted and adjusted longitudinal associations between C1q-fixation, IgG subclass, and IgM levels were also estimated using multilevel modelling. Level-1 error variance estimates from nested regression modeling based on these analyses were used to derive Cohen’s *f*^2^ measures of effect for each IgG subclass and IgM [23]. In these multilevel models, IgG subclass and IgM were estimated as log time-varying fixed factors and their effects unconstrained by time. In addition to these terms, both fixed and random terms for log time were also estimated and a random effect (intercept (level 2)) to account for the dependency C1q-fixation level measurement over-time was also estimated. Statistical inference was assessed at the 5% level.

## Results

### RTS,S-induced anti-CSP IgG in children is predominately IgG1 subclass

**Fig. 1:**
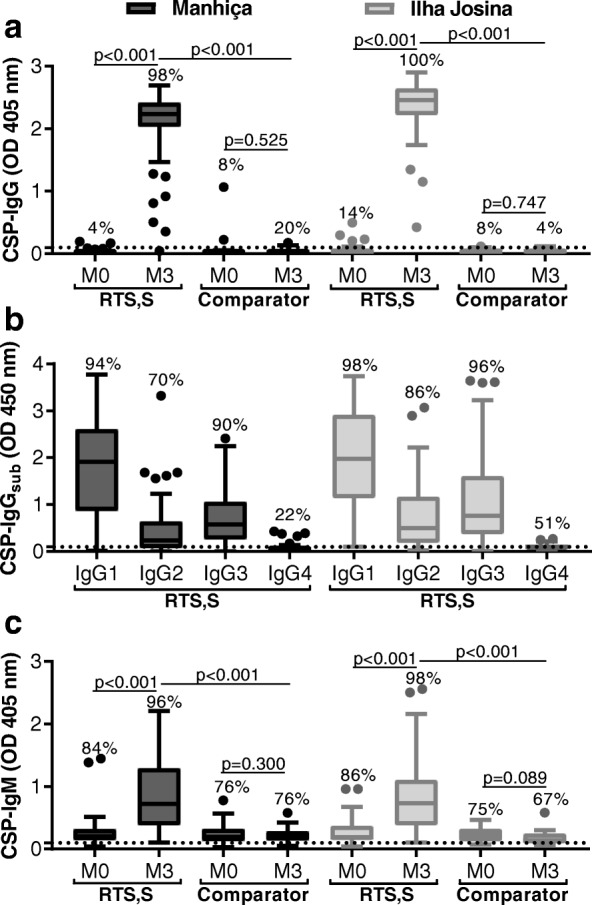
RTS,S vaccine-induced IgG and IgM antibodies to CSP. Children in RTS,S and comparator vaccine groups from Manhiça (black box plots; *N* = 50 and *N* = 25, respectively) and Ilha Josina cohorts (gray box plots; *N* = 49 and *N* = 24, respectively) were tested for IgG (**a**), IgG subclasses (**b**), and IgM (**c**) to CSP. Sera collected at baseline (month 0, M0) and after vaccination (month 3, M3) were tested in duplicate (note that only M3 was tested in **b**), and the mean value was used to generate box plots whereby top, center, and bottom horizontal lines represent the 75th percentile, median, and 25th percentile, respectively; upper and lower whiskers represent the highest and lowest values within 1.5× IQR, respectively; and values that exceed this range are presented as dots. Malaria-naïve negative controls from Melbourne donors were used to calculate the seropositivity cutoff value (dashed lines), and the percentage of individuals above this threshold are shown. Reactivity between paired samples and unpaired samples were compared using Wilcoxon matched-pairs signed-rank test and Mann-Whitney *U* test, respectively

Sera collected at baseline (M0) and 30 days after receiving the final RTS,S vaccination (M3) were tested for total IgG levels to full-length CSP. Reactivity was low at baseline but significantly increased after vaccination with RTS,S in both study cohorts (OD median [IQR]: Manhiça *N* = 50, M0 = 0.020 [0.022], and M3 = 2.231 [0.390], *p* < 0.001; Ilha Josina *N* = 49, M0 = 0.030 [0.039], and M3 = 2.457 [0.425], *p* < 0.001). In contrast, anti-CSP IgG was low at both time points in children who received the comparator non-malaria vaccine (OD median [IQR]: Manhiça *N* = 25, M0 = 0.015 [0.020], and M3 = 0.018 [0.054], *p* = 0.525; Ilha Josina *N* = 24, M0 = 0.029 [0.037], and M3 = 0.038 [0.045], *p* = 0.747) (Fig. 1a).

Children vaccinated with RTS,S (sera collected at M3) were then tested for IgG subclass responses to CSP. Seropositivity was high for IgG1 (94% and 98%) and IgG3 (90% and 96%), followed by IgG2 (70% and 86%) and IgG4 (22% and 51%) in Manhiça and Ilha Josina cohorts, respectively (Fig. 1b). Overall, IgG1 reactivity was highest, followed by IgG3 and IgG2, and only low levels of IgG4 were detected among a minority of subjects. IgG1 demonstrated the strongest correlation with total IgG compared to IgG3 and IgG2, with only a weak correlation for IgG4 (rho = 0.875, rho = 0.689, rho = 615, *p* < 0.001 for all tests; rho = 0.208, *p* = 0.039, respectively) (Additional file 1: Figure S3a).

### RTS,S vaccination induced anti-CSP IgM

We additionally measured IgM levels to CSP at M0 and M3 time points since the induction of IgM may contribute to complement-fixing activity and overall vaccine immunity. To date, there are little data reported on the induction of IgM by RTS,S/AS02_A_. There was a significant increase in IgM reactivity for children vaccinated with RTS,S in both cohorts (OD median [IQR]: Manhiça *N* = 50, M0 = 0.208 [0.178], and M3 = 0.725 [0.912], *p* < 0.001; Ilha Josina *N* = 49, M0 = 0.191 [0.227], and M3 = 0.736 [0.726], *p* < 0.001) but not with comparator vaccine (OD median [IQR]: Manhiça *N* = 25, M0 = 0.194 [0.198], and M3 = 0.194 [0.151], *p* = 0.300; Ilha Josina *N* = 24, M0 = 0.225 [0.200], and M3 = 0.142 [0.157], *p* = 0.089) (Fig. 1c). Furthermore, anti-CSP IgM levels at M3 were significantly higher in the RTS,S vaccine group than the comparator vaccine group for both study sites (*p* < 0.001 for both tests). In all RTS,S-vaccinated children (*N* = 99), anti-CSP IgG and IgM responses at M3 moderately correlated, but many demonstrated IgG-high/IgM-low reactivity. Notably, IgM reactivity at M0 and M3 was weakly correlated (rho = 0.254; *p* = 0.011) (Additional file 1: Figure S3b).

### RTS,S-induced antibodies can fix and activate complement

**Fig. 2:**
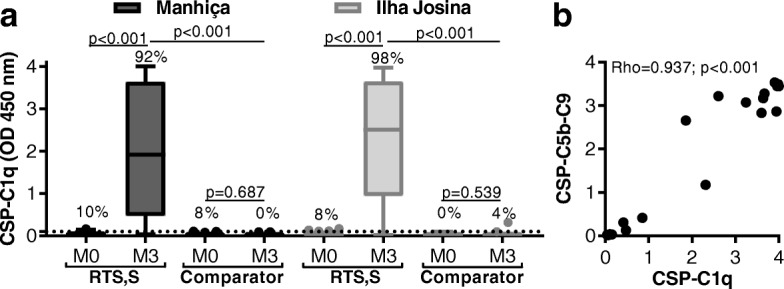
RTS,S vaccine-induced antibodies promote complement fixation to CSP. **a** Children in RTS,S and comparator vaccine groups from Manhiça (black box plots; *N* = 50 and *N* = 25, respectively) and Ilha Josina cohorts (gray box plots; *N* = 49 and *N* = 24, respectively) were tested for C1q-fixation to CSP. Sera collected at baseline (month 0, M0) and after vaccination (month 3, M3) were tested in duplicate, and the mean value was used to generate box plots whereby top, center, and bottom horizontal lines represent the 75th percentile, median, and 25th percentile, respectively; upper and lower whiskers represent the highest and lowest values within 1.5× IQR, respectively; and values that exceed this range are presented as dots. Malaria-naïve negative controls from Melbourne donors were used to calculate the seropositivity cutoff values (dashed lines), and the percentages of individuals above this threshold are shown. Reactivity between paired samples and unpaired samples were compared using Wilcoxon matched-pairs signed-rank test and Mann-Whitney *U* test, respectively. **b** Random selection of children in the RTS,S vaccine group from Manhiça cohort (*n* = 20, M3) were tested for C5b-C9-fixation to CSP, and the mean of duplicates was graphed as scatter plots

We have previously reported that anti-CSP antibodies can activate human complement through recruitment of C1q [17]. In this study, we detected a strong induction of functional antibodies that fix C1q in children vaccinated with RTS,S, which was observed in both study sites (OD median [IQR]: Manhiça *N* = 50, M0 = 0.029 [0.054], and M3 = 1.913 [3.163], *p* < 0.001; Ilha Josina *N* = 49, M0 = 0.013 [0.038], and M3 = 2.508 [2.701], *p* < 0.001). However, this induction of complement-fixing antibodies was not seen with the comparator vaccine (Fig. 2a). RTS,S-induced antibodies could promote activation of the complement cascade, as demonstrated by the presence of complement factors involved in the terminal phase of complement activation that form the membrane attack complex (C5b-C9). Detection of the C5b-C9 complex significantly correlated with C1q-fixation (*n* = 20, rho = 0.937; *p* < 0.001) (Fig. 2b).

### Complement-fixing antibodies target the central repeat and C-terminal regions of CSP

**Fig. 3:**
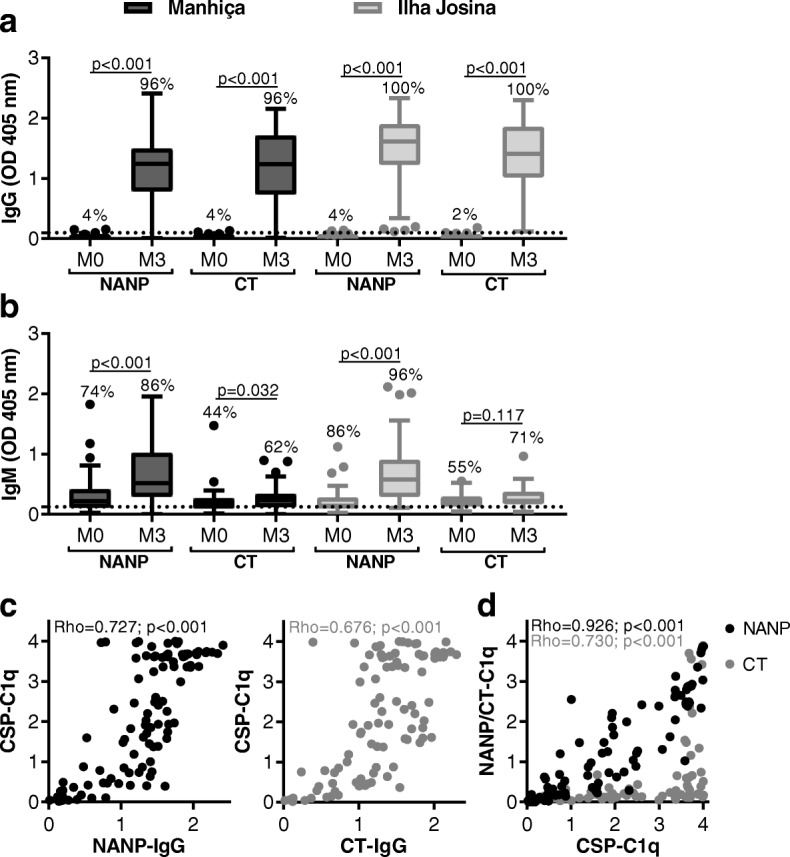
Functional complement-fixing antibodies target the central repeat and C-terminal regions of CSP. Children in RTS,S vaccine group from Manhiça (black box plots; *N* = 50) and Ilha Josina cohorts (gray box plots; *N* = 49) were tested for IgG (**a**) and IgM (**b**) to NANP and CT regions of CSP. Sera collected at baseline (month 0, M0) and after vaccination (month 3, M3) were tested in duplicate, and the mean value was used to generate box plots whereby top, center, and bottom horizontal lines represent the 75th percentile, median, and 25th percentile, respectively; upper and lower whiskers represent the highest and lowest values within 1.5× IQR, respectively; and values that exceed this range are presented as dots. Malaria-naïve negative controls from Melbourne donors were used to calculate the seropositivity cutoff values (dashed lines), and the percentages of individuals above this threshold are shown. Reactivity between paired samples was compared using Wilcoxon matched-pairs signed-rank test. **c**, **d** Children in RTS,S vaccine group from Manhiça and Ilha Josina cohorts (*N* = 99, M3) were tested for C1q-fixation to CSP, NANP, and CT, and the values were plotted compared to IgG reactivity (c) and C1q fixation to CSP, NANP, and CT were correlated (d)

To investigate the targets of functional antibodies induced by RTS,S, we first measured antibody reactivity to NANP and CT antigens, which represented the central repeat and C-terminal regions of CSP, respectively (Fig. 3a, b). Compared to baseline, children vaccinated with RTS,S had significantly higher levels of IgG to both regions of CSP (*p* < 0.001 for all tests), whereas IgM was mostly NANP-specific (NANP, *p* < 0.001 for all tests; CT, *p* = 0.032 and *p* = 0.177 for Manhiça and Ilha Josina cohorts, respectively).

Interestingly, IgG reactivity to NANP and CT significantly correlated with C1q-fixation to full-length CSP, suggesting both regions were involved in the functional activity (rho: NANP = 0.727 and CT = 0.676; *p* < 0.001 for both tests) (Fig. 3c). Indeed, we confirmed that antibodies to each region of CSP could directly fix C1q using specific antigen constructs, although complement-fixing activity was greater against NANP than CT, and correlated more strongly with C1q-fixation to full-length CSP (OD median [IQR]: NANP = 1.25 [2.34] and CT = 0.14 [0.24]; rho: NANP = 0.926 and CT = 0.730; *p* < 0.001 for both tests) (Fig. 3d).

**Fig. 4:**
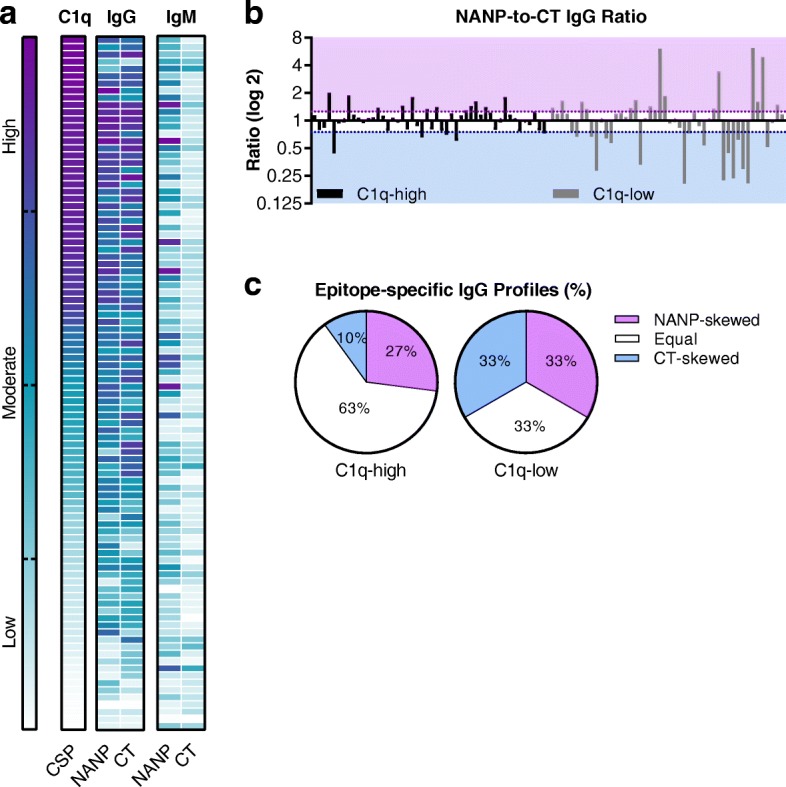
High variability among RTS,S vaccine-induced IgG targeting the central repeat and C-terminal regions of CSP. Children in RTS,S vaccine group from Manhiça and Ilha Josina cohorts (*N* = 99, 3 M) were tested for IgG to NANP and CT and C1q-fixation to CSP, and the values were used for the following analysis. **a** Heat map of children arranged in descending (top to bottom) order of C1q-fixation (left), corresponding IgG to NANP and CT (middle), and for comparison IgM to NANP and CT (right). **b** Variability between epitope specificity was quantified by calculating the ratio of NANP-to-CT IgG. Children with low variability were considered to have equal reactivity to NANP and CT (ratio between 0.75 and 1.25 shown in white), and children exceeding this range were considered to have a NANP- or CT-skewed response (ratio > 1.25 shown in purple and ratio < 0.75 shown in blue, respectively). Children were arranged in descending order of C1q-fixation (left to right), whereby those in the top quantile are in black bars (C1q-high, OD range 2.03 to 4.00) and those in the bottom quantile are in gray bars (C1q-low, OD range 0.02 to 1.97). **c** Pie charts representing the percentage of children with the following epitope-specific IgG profiles from C1q-high and C1q-low groups: NANP-skewed, equal, and CT-skewed

We then compared antibody responses between children who had high or low levels of complement-fixing antibodies to CSP (defined based on the median: high *N* = 50, OD range 2.03 to 4.00; low *N* = 49, OD range 0.02 to 1.97). Notably, children with high C1q-fixing antibodies had substantially higher levels of IgG to both NANP and CT and higher levels of IgM to NANP (IgG: NANP *p* < 0.001, CT *p* < 0.001; IgM: NANP *p* < 0.001, CT *p* = 0.038) (Additional file 1: Figure S4). We further examined the epitope specificity using heat maps and found high variability among individuals with respect to IgG and IgM levels to the different regions (Fig. 4a). We explored different approaches to identify potential influencing factors for complement fixation activity. Linear regression for anti-NANP IgG with the additional inclusion of anti-CT IgG did not substantially improve the fit of the model overall compared to IgG to NANP alone, and we found no significant linear relationship between epitope-specific IgM by linear regression (Additional file 1: Table S1). Given the observed variability in IgG responses to the NANP and CT regions, we calculated the ratio of NANP-to-CT IgG, which was used to categorize children in equal, NANP-skewed, or CT-skewed IgG epitope profiles (defined by ratios of 0.75 to < 1.25, > 1.25, and < 0.75, respectively) (Fig. 4b). Interestingly among the high complement-fixing group, a greater proportion had relatively equal levels of IgG to both NANP and CT (63%), while some were NANP-skewed (27%) and fewer CT-skewed (10%). For children with low C1q-fixing antibodies, all three epitope profiles were equally observed (33% each) (Fig. 4c). This suggests that IgG reactivity to both regions may be favorable for C1q-fixation to full-length CSP.

### Poor induction of functional antibodies in children with higher malaria exposure and blood-stage immunity

**Fig. 5:**
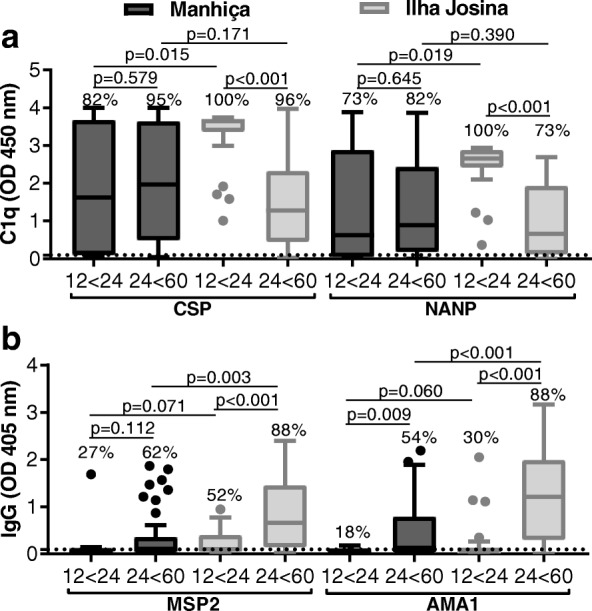
Relationship between age and immunity. Children in RTS,S vaccine group from Manhiça (black box plots) and Ilha Josina cohorts (gray box plots) were categorized into younger (12 to 24 months; Manhiça *n* = 11 and Ilha Josina *n* = 23, respectively) and older (24 to 60 months; Manhiça *n* = 39 and Ilha Josina *n* = 26, respectively) age groups. Sera collected after vaccination (month 3, M3) were tested for C1q-fixation to CSP and NANP (**a**) and IgG-reactivity to blood-stage antigens MSP2 and AMA1 (**b**). Samples were tested in duplicate, and the mean value was used to generate box plots for samples stratified by age group. Top, center, and bottom horizontal lines represent the 75th percentile, median, and 25th percentile, respectively; upper and lower whiskers represent the highest and lowest values within 1.5× IQR, respectively; and values that exceed this range are presented as dots. Malaria-naïve negative controls from Melbourne donors were used to calculate the seropositivity cutoff values (dashed lines), and the percentages of individuals above this threshold are shown. Reactivity between unpaired samples was compared using Mann-Whitney *U* test

To evaluate the potential effect of malaria exposure on the induction of complement-fixing antibodies, we compared RTS,S-induced functional antibodies (sera collected at M3) in younger and older age groups (12–24 and 24–60 months, respectively) and between our two different study sites (Additional file 1: Figure S5a). Children in the Manhiça cohort demonstrated no relationship between age and C1q-fixation to CSP (younger *n* = 11 and older *n* = 39, *p* = 0.579; rho = − 0.121, *p* = 0.403). For the Ilha Josina cohort, which had a substantially higher level of malaria transmission than Manhiça, younger children had significantly higher levels of C1q-fixing antibodies (younger *n* = 23 and older *n* = 26, *p* < 0.001), and age negatively correlated with C1q-fixation (rho = − 0.575, *p* < 0.001). Similar age associations were also observed for C1q-fixation to NANP (Fig. 5a) as well as anti-CSP IgG and IgG subclass responses. However, anti-CSP IgM was comparable between the age groups for the Ilha Josina cohort but was interestingly higher in older children for the Manhiça cohort (Additional file 1: Figures S5b-d).

The Ilha Josina study site had a higher level of malaria transmission intensity than Manhiça, indicating that age associations may be related to malaria exposure. To investigate, we measured antibody responses to well-characterized blood-stage antigens, MSP2 and AMA1, which are established biomarkers of malaria exposure [24] (Fig. 5b). The relationship between age and blood-stage antibodies was weak for the Manhiça cohort (MSP2 *p* = 0.112 and AMA1 *p* = 0.009; correlation with age, MSP2 rho = 255, *p* = 0.074, and AMA1 rho = 0.302, *p* = 0.033) but strong for the Ilha Josina cohort (MSP2 *p* < 0.001 and AMA1 *p* < 0.001; correlation with age, MSP2 rho = 0.595, *p* < 0.001, and AMA1 rho = 0.605, *p* < 0.001). Furthermore, in the Ilha Josina cohort, C1q-fixation to CSP negatively correlated with IgG to MSP2 and AMA1 (rho = − 0.453, *p* = 0.001; rho = − 0.443, *p* = 0.002, respectively). Collectively, these data suggest that increased malaria exposure negatively impacts the induction of functional immunity by RTS,S immunization.

### Functional antibodies are poorly sustained after RTS,S vaccination

**Fig. 6:**
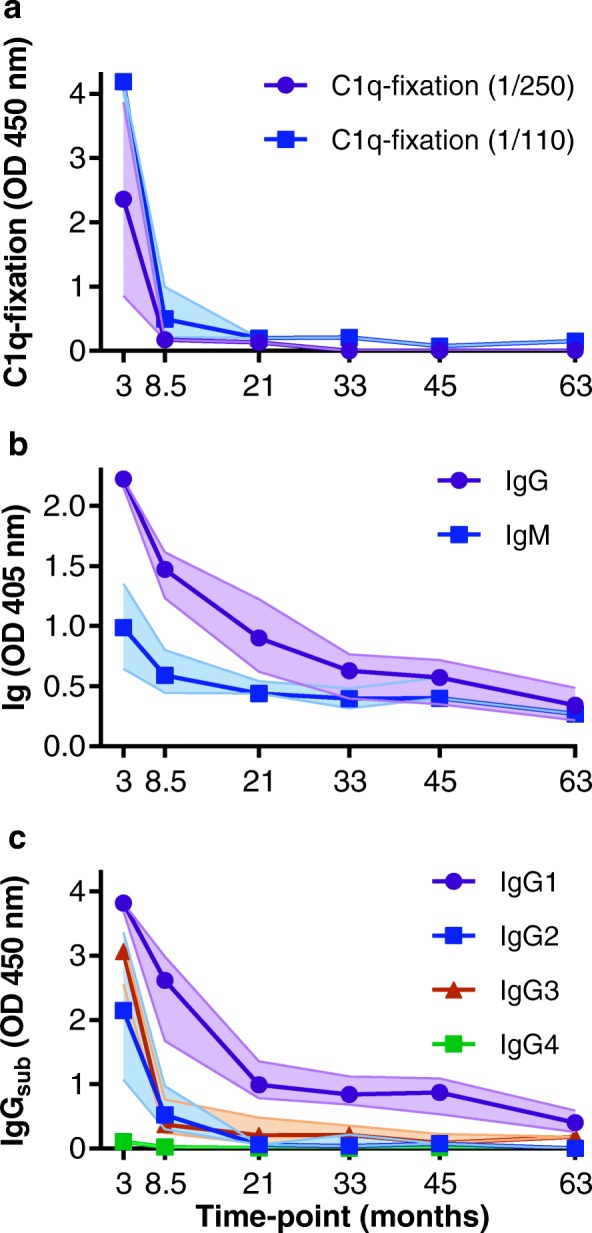
RTS,S vaccine-induced immunity declines over time. A random selection of children vaccinated with RTS,S (Manhiça cohort, *n* = 30) was tested for C1q-fixation (**a**), IgG/IgM (**b**), and IgG subclasses (**c**) to CSP at months 3, 8.5, 21, 33, 45, and 6. Note that due to low reactivity, C1q-fixation was re-tested at a higher dilution of 1/110, in addition to 1/250, to confirm results. Samples were tested in duplicate, and the median and 95% CI of the median from each time point group are shown by the symbol and shaded area, respectively

To assess long-term RTS,S-induced immunity, we examined a random selection of children from the Manhiça cohort (*n* = 30) with samples available at months 3, 8.5, 21, 33, 45, and 63 (5-year follow-up). Maximal C1q-fixation responses were observed at M3, which then significantly declined by M8.5 (OD median, 2.363 and 0.174, respectively; *p* < 0.001) (Fig. 6a and Additional file 1: Figure S6). Sera were re-tested at a higher concentration (1/110 dilution), and a similar result was obtained. Interestingly, a small number of individuals did maintain complement-fixing activity for a longer period (Additional file 1: Figure S6). Antibody concentration is known to effect complement activity, and therefore, the decline in C1q-fixation responses may have been due to antibody levels being below the required functional threshold (Additional file 1: Figure S7).

IgG and IgM responses to CSP also waned over time, although this did not appear as marked as the loss of C1q-fixing antibodies (Fig. 6b). Given that IgG subclass strongly influences antibody function, we examined the decay of each IgG subclass response with respect to the decline in functional C1q-fixing activity (Fig. 6c). IgG1 and IgG3 responses both declined substantially over time, consistent with the decline in complement-fixing antibodies.

### Modeling the decay of complement-fixing antibodies

Determining the decay rate of specific antibody types is important for understanding the duration of vaccine-induced immunity and informs how this may be improved in the future. Therefore, we developed methods to estimate the decay rates of complement-fixing antibodies, IgG subclasses, and IgM, using latent growth curve modeling (Table 1). Antibody decay followed a largely exponential decay, and a log of the data was used to normalize the data. Descriptively, the rate of decline of IgG1, IgG2, and IgG3 was similar, whereas IgM decay was slower. IgG4 was estimated to have a slow decay rate; however, the levels of IgG4 were very low overall. The rate of decay of complement-fixing antibodies was higher than that seen for total IgG. The half-life of complement-fixing antibodies was relatively short (5.2 [3.8–6.5] months) compared to that of IgG1 (8.4 [6.0–10.8] months), IgG2 (10.8 [5.70–15.9] months), and IgG3 (8.6 [6.2–10.9] months) subclass responses.

**Table 1:** Associations between complement fixation, antibody responses, and time using latent growth curve modeling (*n* = 30)

Outcome^a^	*b*^b^ (s.e.)	95% CI	*p* value^c^
log C1q	− 0.42 (0.03)	− 0.49, − 0.36	< 0.001
log IgG1	−0.33 (0.02)	− 0.37, − 0.28	< 0.001
log IgG2	− 0.30 (0.02)	− 0.33, − 0.26	< 0.001
log IgG3	− 0.32 (0.02)	− 0.36, − 0.28	< 0.001
log IgG4	− 0.03 (0.004)	− 0.04, − 0.02	< 0.001
log IgM	− 0.12 (0.02)	− 0.16, − 0.09	< 0.001

^a^Effects for each factor represent separate linear mixed modeling (LMM) analyses where the natural log of C1q-fixation and each antibody response is regressed on the natural log of time (per month). These LMMs applied a random intercept for study participant, random slope for log time with an unstructured covariance parameter specified for the correlation between the random effects

^b^Regression coefficient (*b*) and standard error (s.e.) represent the percent change in C1q-fixation or antibody response for a percent increase in time

^c^Probability values based on Wald statistics

To better understand the factors contributing to the decline in complement-fixing antibodies over time, we developed a model to determine which antibodies most strongly correlated with declining complement-fixing activity using the repeated measures data. We undertook nested multivariable mixed modeling to estimate the unique variance of complement-fixing antibodies by each antibody response type (Table 2). The analysis indicated that longitudinal complement-fixation responses were most strongly independently associated with IgG1 (*f*^2^ = 0.38, Wald *χ*^2^(2) = 55.3; *p* < .001) and there was also a significant correlation with IgG3 (*f*^2^ = 0.18, Wald *χ*^2^(2) = 29.5; *p* < .001). There was no significant association with IgG2, IgG4, or IgM.

**Table 2:** Associations between antibody responses and complement fixation activity over time using linear mixed modeling (*n* = 30)

Factor^a^	*b*^b^ (s.e.)	95% CI	*p* value^c^	*f* ^2d^
IgG1				0.38
Linear term	1.10 (0.16)	0.78, 1.41	< 0.001	
Interaction term	− 0.28 (0.05)	− 0.38, − 0.18	< 0.001	
IgG2				0.02
Linear term	0.11 (0.13)	− 0.14, 0.37	0.388	
Interaction term	− 0.01 (0.05)	− 0.10, 0.08	0.805	
IgG3				0.18
Linear term	0.54 (0.15)	0.24, 0.83	< 0.001	
Interaction term	− 0.07 (0.05)	− 0.18, 0.03	0.177	
IgG4				0.02
Linear term	0.21 (0.86)	− 1.47, 1.90	0.802	
Interaction term	0.07 (0.31)	− 0.53, 0.68	0.808	
IgM				0.03
Linear term	− 0.21 (0.18)	− 0.57, 0.38	0.233	
Interaction term	− 0.02 (0.07)	− 0.11, 0.15	0.792	

^a^Linear mixed modeling (LMM) analyses where the natural log of C1q-fixation was regressed on the natural log of antibody responses (linear term) conditioning on other antibody response factors to provide an independent association. This LMM model included a term for the natural log of time (not shown) and relaxed the constraint of a consistent association between C1q-fixation and antibody responses across time (interaction term). The LMMs applied a random intercept for study participant and random slope for time

^b^Regression coefficient (*b*) and standard error (s.e.) represent the percent change in participant C1q-fixation level for a percent increase in antibody responses

^c^Probability values based on Wald statistics

^d^Cohen’s *f*^2^ represents the ratio of the unique variance explained by a specific antibody response factor to the variance explained by an intercept-only model. Higher values indicate a stronger effect

## Discussion

Knowledge of the induction and maintenance of functional immune responses by RTS,S is valuable for strategically improving vaccine design and delivery to generate a highly efficacious and longer-lasting malaria vaccine. Here, we investigated RTS,S-induced antibody responses in children and present new evidence of a functional mechanism that may contribute to vaccine-induced immunity. We show that RTS,S-induced anti-CSP antibodies were predominantly IgG1, and there was also a substantial induction of IgG3, IgG2, and IgM. Antibodies among vaccinated children could strongly fix complement on the whole, but functional responses did vary among individuals. Our data suggest that antibodies targeting both the central repeat and C-terminal regions of CSP are important for complement fixation. Furthermore, functional antibodies were poorly induced in older children with greater malaria exposure, and functional antibodies were poorly maintained after vaccination. This decline in complement-fixing antibodies was largely due to a decline in IgG1 and to a lesser extent IgG3. These findings highlight the diversity of RTS,S-induced immune responses in malaria-exposed populations, demonstrate a new functional mechanism of vaccine-induced antibodies, and shed some light on the maintenance and decay of vaccine immunity. Our findings suggest that generating higher levels of complement-fixing antibodies among a greater proportion of children, and inducing responses that are durable over time, may contribute to greater vaccine efficacy and durability.

IgG1 and IgG3 subclasses have the highest complement-fixing activity, whereas IgG2 and IgG4 have little or no activity [25]. RTS,S/AS02_A_ strongly induced anti-CSP IgG1 followed by IgG3 and IgG2 in children aged 1–4 years. Similar findings showing a predominant IgG1 and IgG3 response were recently reported for a subset (*n* = 195) of subjects from the phase III RTS,S/AS01_B_ vaccine trial of infants and young children (6–12 weeks and 5–17 months). A higher ratio of cytophilic antibodies (IgG1 and IgG3) to non-cytophilic antibodies (IgG2 and IgG4) was associated with vaccine-induced protection [8]. Therefore, we propose that one way in which RTS,S-induced cytophilic antibodies may confer protection is by interacting with human complement, a mechanism that has been previously associated with protection in studies of naturally acquired immunity to sporozoites in children [17]. The AS01_B_ and AS02_A_ adjuvants both contain TLR4 agonist MPL and the saponin QS-21, but AS01_B_ is a liposome-based adjuvant whereas AS02_A_ is an oil-in-water emulsion [26]; RTS,S administered with either adjuvant appears to induce similar IgG subclass profiles in malaria-exposed children. In the phase III study, post-vaccination levels of IgG2 and IgG4, but not IgG1 and IgG3, to CSP were significantly higher in the high malaria transmission site compared to the low malaria transmission site [8]. It is noteworthy that studies in malaria-naïve adults found RTS,S to predominately induce IgG1 and IgG2 only [27–29], which demonstrates that immunogenicity can substantially differ in malaria-exposed populations compared to malaria-naïve individuals in non-endemic countries. Such differences are poorly understood but require a greater understanding, particularly because IgG3 is a potent mediator of complement fixation and activation. The use of adjuvants or vaccine regimens that increase IgG3 and reduce IgG2 induction is likely to lead to responses with greater complement-fixing activity and potentially greater efficacy.

Complement activity can also be mediated by IgM antibodies. Interestingly, anti-CSP IgM seropositivity was moderate at baseline, although functional C1q-fixation responses were only apparent after RTS,S vaccination when IgM (and IgG) had significantly increased. Modeling the decay of antibodies over time suggests IgM is not a major mediator of complement-fixing activity, and this was instead largely mediated by IgG1 and IgG3. The role of IgM in immunological memory is not well established, but a recent study demonstrated that IgM memory B cells were long-lived and involved in secondary responses using a murine model of malaria [30], and IgM responses remain present even in those with extensive malaria exposure [31].

Complement activity is also influenced by epitope-specificity [32–34]. We found that RTS,S-induced antibodies were most potent at fixing complement to the central repeat region of CSP, but antibodies to the C-terminal region could also fix complement. Interestingly, children with higher complement-fixing antibodies tended to have equal levels of IgG to both CSP regions, whereas those with low functional antibodies were more frequently epitope-skewed. Therefore, antibodies to both regions may better promote immune complex formation that leads to complement fixation and activation. Overall, there were highly diverse IgG epitope profiles among children. In field evaluations of RTS,S, only IgG to the central repeat region have been typically measured and reported. This is likely because RTS,S-induced antibodies to the central repeat region, but not the C-terminal region, show some association with protection in studies of healthy malaria-naïve adults [10, 29, 35–37]. However, the relationship between protection and repeat-specific antibodies has been inconsistently reported in field evaluations of malaria-exposed children and infants [18, 38, 39]. A recent study found that antibodies to both regions of CSP, that were of a specific IgG subclass, were associated with protection in the phase III clinical trial of RTS,S/AS01_B_ [8]. Additionally, the central repeat region is considered immunodominant as it is a major target of antibodies induced by whole irradiated attenuated sporozoite vaccines and natural malaria exposure [40, 41], and repeat-specific antibodies can block sporozoite infectivity in vitro [42]. However, antibodies to non-repeat regions are also naturally acquired and have demonstrated inhibitory activity in vitro [43–47]. Taken together, our data support the importance of antibodies to the central repeat region of CSP, but also encourage further investigation of antibody responses to the C-terminal region, particularly because repeat-specific antibodies alone are a poor correlate of protection.

RTS,S-induced antibodies strongly fixed complement to CSP, but overall functional activity among vaccinated children markedly declined by M8.5. Although total IgG remained moderate at this time, reactivity may have dropped below the threshold required to detect functional activity using our methods, particularly because antibody density is known to influence complement activity, as multiple C1q monomers (six in total) bind neighboring IgG molecules to initiate the classical complement pathway [32]. Our statistical modeling indicated that the rapid decay of complement-fixing antibodies was mostly explained by the decline in IgG1 and to a lesser extent IgG3, likely because both subclasses can potently fix complement and because IgG1 was the predominant subclass induced by vaccination. Perhaps, if RTS,S induced greater levels of IgG3 that were above the antibody threshold needed for functional activity and were more durable, complement-fixing antibody responses may have better maintained after vaccination. Including measures over time enabled a better understanding of the relationships between specific antibody types and complement fixation than analysis performed at a single cross-sectional time point. While the decay post-immunization of IgG to the central repeat region has been reported [48], there are no data on the decay of functional antibodies, IgG subclasses, or IgM. Additionally, no malaria vaccine studies have reported the relationship between IgG or IgM and functional antibodies over time. Therefore, our new statistical methods developed for this study may be valuable for understanding these responses in other RTS,S trials and studies of other malaria vaccines. It is possible that functional antibodies, IgG subclasses, and IgM have different responses and kinetics after booster immunizations. The decay of complement-fixing antibodies was broadly consistent with the decline in RTS,S vaccine efficacy for this trial. Statistical analyses estimated that initial efficacy against clinical malaria was ~ 30% in the first 4–5 months but quickly waned to limited efficacy 18–30 months after vaccination. In our studies, some children retained significant complement-fixing antibodies at M8.5, which may contribute to some continuing vaccine efficacy. Our findings suggest these questions warrant investigation in the phase III trials to better understand the efficacy and durability of RTS,S.

The present study was conducted in a malaria-endemic setting, and therefore, children will have varying degrees of malaria exposure and naturally acquired immunity. Older children in the higher malaria transmission site, Ilha Josina, had significantly lower levels of complement-fixing antibodies. This was also observed for total IgG to CSP, but the effect was not as pronounced. Older children also had higher levels of antibodies to blood-stage antigens, and this inversely correlated with vaccine-induced anti-CSP antibodies, which has been previously reported [49, 50]. Interestingly, these trends were only clear in the Ilha Josina study site, where transmission intensity was approximately tenfold higher than the Manhiça site. These findings suggest that age and higher levels of malaria exposure negatively impact on the induction of functional antibodies by the RTS,S vaccine. This is a significant finding that has implications for generating immunogenic and efficacious malaria vaccines in high transmission zones. The biological basis for this effect is not known but could be due to T or B cell exhaustion from repeated exposure [51], or naturally acquired immunity could be interfering with the induction of vaccine immunity. Understanding this issue is a priority for further research.

## Conclusions

We have provided a detailed characterization of RTS,S-induced antibody responses in malaria-exposed children and identified a new functional antibody activity induced by RTS,S. This is an important finding as recent studies demonstrated that complement fixation by antibodies could inhibit sporozoite traversal and lead to sporozoite cell death [16, 17], suggesting that RTS,S-induced immunity may function, in part through complement fixation. Our data demonstrates highly diverse patterns of epitope specificity among children and raises the potential role of the C-terminal region of CSP in functional immunity, in addition to antibodies targeting the NANP region. This finding calls for further examination of how antibodies to the C-terminal region may co-operatively promote antibody function, such as complement activation. Complement-fixing antibodies were generally relatively short-lived and were less effectively induced in older children with higher malaria exposure, which could be important for vaccine implementation in locations with high malaria transmission intensity and should be further evaluated. Furthermore, we developed statistical methods to investigate antibody decay and relationships over time post-vaccination; these approaches could be highly valuable in future vaccine trial evaluation, especially as a tool to investigate the determinants of vaccine longevity and inform strategies to improve this. Our findings support future investigation of the large phase III trials to determine whether complement fixation is a valuable correlate of protective immunity and advance the development of more efficacious and long-lasting vaccines.

## Additional file

Additional file 1: Induction and decay of functional complement-fixing antibodies by the RTS,S vaccine in children and a negative impact of malaria exposure supplementary material. Supplementary equation 1. Supplementary methods for recombinant C-terminal region of CSP (**Figure S1.**). Supplementary methods for C1q-fixation assay (**Figure S2.**). **Figure S3.** RTS,S predominately induces anti-CSP IgG1 and some IgG3, IgG2, and IgM antibodies. **Figure S4.** Epitope specificity of children with high or low C1q-fixing antibodies. **Figure S5.** Younger and older children in Manhiça and Ilha Josina cohorts. **Figure S6.** Functional complement-fixing antibodies decline over time. **Figure S7.** Antibody concentration and functional C1q-fixation responses. Table S1. Linear regression between epitope-specific IgG and C1q-fixation to CSP, induced by vaccination with RTS,S (*N* = 99). (DOCX 2573 kb)

## Abbreviations

ABTS: 2,2′-Azino-bis(3-ethylbenzothiazoline-6-sulfonic acid)
AIC: Akaike Information Criterion
AMA1: Apical membrane antigen 1
BIC: Bayesian Information Criterion
CSP: Circumsporozoite protein
CT: Construct representing the C-terminal region of CSP
ELISA: Enzyme-linked immunosorbent assay
HRP: Horseradish peroxidase
IgG: Immunoglobulin G
IgM: Immunoglobulin M
IQR: Interquartile range
M0: Month 0
M3: Month 3
MSP2: Merozoite surface protein 2
NANP: Peptide representing the central repeat region of CSP
OD: Optical density
PBS: Phosphate-buffered saline
RT: Room temperature
TMB: Tetramethylrhodamine
