# Supplementary material for: Induction and decay of functional complement-fixing antibodies by the RTS,S malaria vaccine in children, and a negative impact of malaria exposure
Source: BMC Med. 2019 Feb 25;17:45. doi: 10.1186/s12916-019-1277-x (PMC6388494; doi:10.1186/s12916-019-1277-x)
Supplement: Supplementary file 1 — Induction and decay of functional complement-fixing antibodies by the RTS,S vaccine in children and a negative impact of malaria exposure supplementary material. Supplementary equation 1. Supplementary methods for recombinant C-terminal region of CSP (Figure S1.). Supplementary methods for C1q-fixation assay (Figure S2.). Figure S3. RTS,S predominately induces anti-CSP IgG1 and some IgG3, IgG2, and IgM antibodies. Figure S4. Epitope specificity of children with high or low C1q-fixing antibodies. Figure S5. Younger and older children in Manhiça and Ilha Josina cohorts. Figure S6. Functional complement-fixing antibodies decline over time. Figure S7. Antibody concentration and functional C1q-fixation responses. Table S1. Linear regression between epitope-specific IgG and C1q-fixation to CSP, induced by vaccination with RTS,S (N = 99). (DOCX 2573 kb) [file 12916_2019_1277_MOESM1_ESM.docx]

# Induction and decay of functional complement-fixing antibodies by the RTS,S vaccine in children, and a negative impact of malaria exposure

## Additional file 1

## Authors and affiliations:

Liriye Kurtovic^1,2^, Paul A. Agius^1,3^, Gaoqian Feng^1^, Damien R. Drew^1^, Itziar Ubillos^4^, Jahit Sacarlal^5,6^, John J. Aponte^4,5^, Freya J. I. Fowkes^1,3,7^, Carlota Dobaño^4,5^, James G. Beeson^1,2,8,9*^

^1^Burnet Institute, Melbourne, Australia. ^2^Department of Immunology and Pathology, Monash University, Melbourne, Australia. ^3^Department of Epidemiology and Preventative Medicine, Monash University, Melbourne, Australia. ^4^ISGlobal, Hospital Clínic, Universitat de Barcelona, Barcelona, Catalonia, Spain. ^5^Centro de Investigação em Saúde de Manhiça, Maputo, Mozambique. ^6^Faculdade de Medicina, Universidade Eduardo Mondlane (UEM), Maputo, Mozambique. ^7^Centre for Epidemiology and Biostatistics, Melbourne School of Population and Global Health, The University of Melbourne, Melbourne, Australia. ^8^Department of Microbiology, Monash University, Clayton, Australia. ^9^Department of Medicine, The University of Melbourne, Parkville, Australia.

*Corresponding author: 85 Commercial Road, Melbourne, Australia, 9282 2111, beeson@burnet.edu.au

## Supplementary equation 1

$y_{ij}= \beta_{1}+ \beta_{2}log({t)}_{ij}+ \zeta_{1j}+ \zeta_{2j}t_{ij +} \epsilon_{ij}$ with $\zeta_{nj}$ ~ N(0,$\sigma^{2}$) (1)

Where $\beta_{1}$ is the mean baseline log C1q-fixation and log IgG subclass and IgM level, $\beta_{2}log({t)}_{ij}$ the term representing the functional form of time for person j at occasion i, $\zeta_{1j}$the random effect (i.e. intercept) for between-person variability in baseline log C1q-fixation and log IgG subclass and IgM level , $\zeta_{2j}t_{ij}$ the random effect (i.e. coefficient) for between-person variability in log C1q-fixation and log IgG subclass and IgM level change across time and $\epsilon_{ij}$ the person-specific between-response residual.

## Supplementary methods for recombinant C-terminal region of CSP

### Sequence selection and modification

The C-terminal region (CT) construct was based on *P. falciparum* CSP 3D7 sequence (XP_001351122) between amino acids 273-383; note the GPI anchor motif was disrupted. To mediate protein secretion and purification (via nickel resin chromatography), a signal peptide for Tissue Plasminogen Activator (TPA) protein followed by a 6-histidine (6His) tag was fused to the N-terminus. Cleavage of the TPA signal peptide from the secreted from of the recombinant C-terminal CSP protein immediately N-terminal to the 6His tag was confirmed using SignalP 4.1 (http://www.cbs.dtu.dk/services/SignalP/)

### Generation of protein expression vector

The protein sequence was used to generate DNA sequences that were codon optimized for mammalian expression and then synthesized (GeneArt). The synthetic genes were supplied in a Puc vector, and then cloned into pcDNA 3.1+ using *Nhe*1 and *Bam*H1 restriction sites. The final plasmids was quantified and used to transfect HEK293F cells.

### HEK293F cell culture and transfection

HEK293 FreestyleTM cells (Thermo Fisher Scientific) were cultured as described by the manufacturer. Briefly, HEK293 cells were cultured in Erlenmeyer shaker flasks (125 ml, Corning) with FreeStyle™ 293 Expression Medium (Thermo Fisher Scientific) at 37°C, 8% CO_2_ at 135 rpm on an orbital shaker. Cells were counted using the trypan blue (0.4%; Thermo Fisher Scientific) cell exclusion method using CountessTM Cell Counting Chamber Slides (Thermo Fisher Scientific) and the CountessTM automated cell counter (Thermo Fisher Scientific). HEK293F cells were transfected for protein expression as described by the manufacturer (Thermo Fisher Scientific) with minor alterations. On the day of transfection, cells were centrifuged (700 RCF, 10 minutes, 4°C) and resuspended in HEK293F expression media with 1:100 antibiotic/anti-mycotic solution (Thermo Fisher Scientific) at a final density of 1x10^6^ cells/ml. For a 30 ml transfection, 90 µl of Polyethylenimine transfection reagent (25 kDA linear; Polysciences; stock 1 mg/ml) was added to 0.6 ml of OptiProTM Serum Free Medium (Thermo Fisher Scientific) and incubated for 5 minutes. This was then added to the DNA solution (30 µg of purified plasmids and 0.6 ml of OptiProTM Serum Free Medium (Thermo Fisher Scientific)) and incubated for 10 minutes at room temperature. This final solution was added to the cells, and returned to the orbital shaker and incubator. The next day, Lupin (1:40 of 20% w/v, Biotech Solabia) and Pluronic acid F-68 (1:100 of 10 % w/v) (Thermo Fisher Scientific) was added. Expressed protein were harvested 6 days post-transfection by centrifuging cells (700 RCF, 10 minutes) to collect supernatant that was then filtered (0.2µm membrane) and stored at 4°C until purification.

### Protein purification and dialysis

The harvested media containing the expressed CT was passed over nickel resin columns (Life Technologies), washed with 20mM imidazole/PBS (Sigma-Aldrich), and bound proteins eluted in several 1 ml fractions in 500mM imidazole /PBS. Eluted fractions were tested for the presence of protein by spectroscopy and SDS-PAGE. Fractions containing protein were pooled, filter sterilized, dialyzed into sterile PBS, and adjusted to a concentration of 1 mg/ml via centrifugation using 10,000MW cut-off filters. Protein size and purity was confirmed by SDS-PAGE (**Figure S1**).

## Supplementary methods for C1q-fixation assay

### Generation of rabbit anti-C1q IgG

Rabbits received three immunizations of purified human C1q (Millipore, 200 µg/dose), whereby the first dose was administered in complete Freud’s adjuvant and the last two in incomplete Freud’s adjuvant. Rabbit immunizations were approved by the Animal Ethics Committee of the Walter and Eliza Hall Institute. After all three immunizations, polyclonal IgG was purified from the anti-serum at the Walter and Eliza Hall Institute.

### Validation of detection antibodies

The rabbit anti-C1q IgG was tested for specificity and sensitivity by Western blot. Human serum depleted of C5 or C1q (both 1/10 dilution, Millipore) were processed for SDS-PAGE under reducing conditions, and blotted onto nitrocellulose membrane. The membrane was blocked in 10% skim milk in PBS-Tween20 0.05% (v/v), and probed with rabbit anti-C1q antibodies (commercial and in-house) followed by HRP conjugated detection antibodies, with membrane washing in between (three 5 minute washes in PBS-Tween20 0.05%). Protein bands were detected using chemiluminescent substrate and autoradiography film. The in-house rabbit anti-C1q IgG demonstrated strong specificity and sensitivity to C1q (alpha chain ~29 kDa and beta chain ~27 kDa), which was similar to that of the commercially available detection antibody (**Figure S2a**),

The goat anti-rabbit IgG HRP detection antibody (Millipore) was tested against IgG from various species by standard ELISA. The antibody only bound to rabbit IgG, and importantly did not cross-react with human IgG, and was therefore considered to be highly specific (**Figure S2b**).

## Supplementary figures and tables

mdamkrglccvlllcgavfvsashhhhhhNKNNQGNGQGHNMPNDPNRNVDENANANSAVKNNNNEEPSDKHIKEYLNKIQNSLSTEWSPCSVTCGNGIQVRIKPGSANKPKDELDYANDIEKKICKMEKCSSVFNVVNS


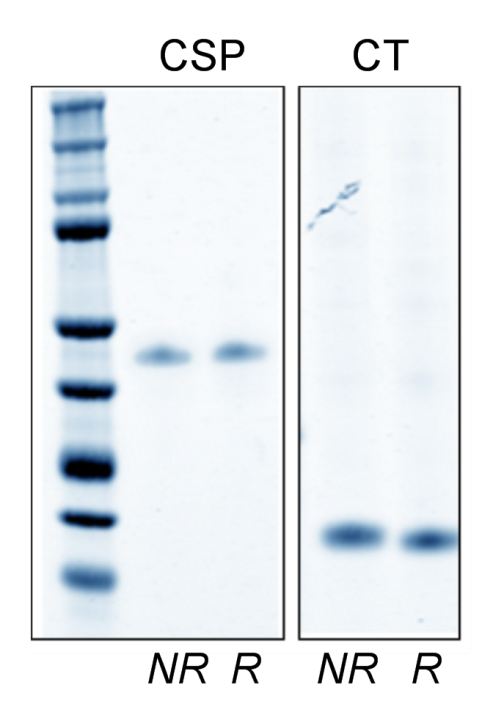


**Figure S1. Sequence and purity of recombinant C-terminal region of CSP.** Top panel: amino acid sequence: the Tissue Plasminogen Activator signal peptide is shown in blue, the 6-histidine tag is shown in green and the C-terminal region (amino acids 273-383) is shown in black. Bottom panel: Purity of full length CSP (Sanaria) and purified CT under non-reduced (NR) and reduced (R) conditions, by SDS-PAGE stained using Colloidal Blue (1 μg/well). Note that proteins were loaded into wells 2, 3, 8 and 9 of the same gel.


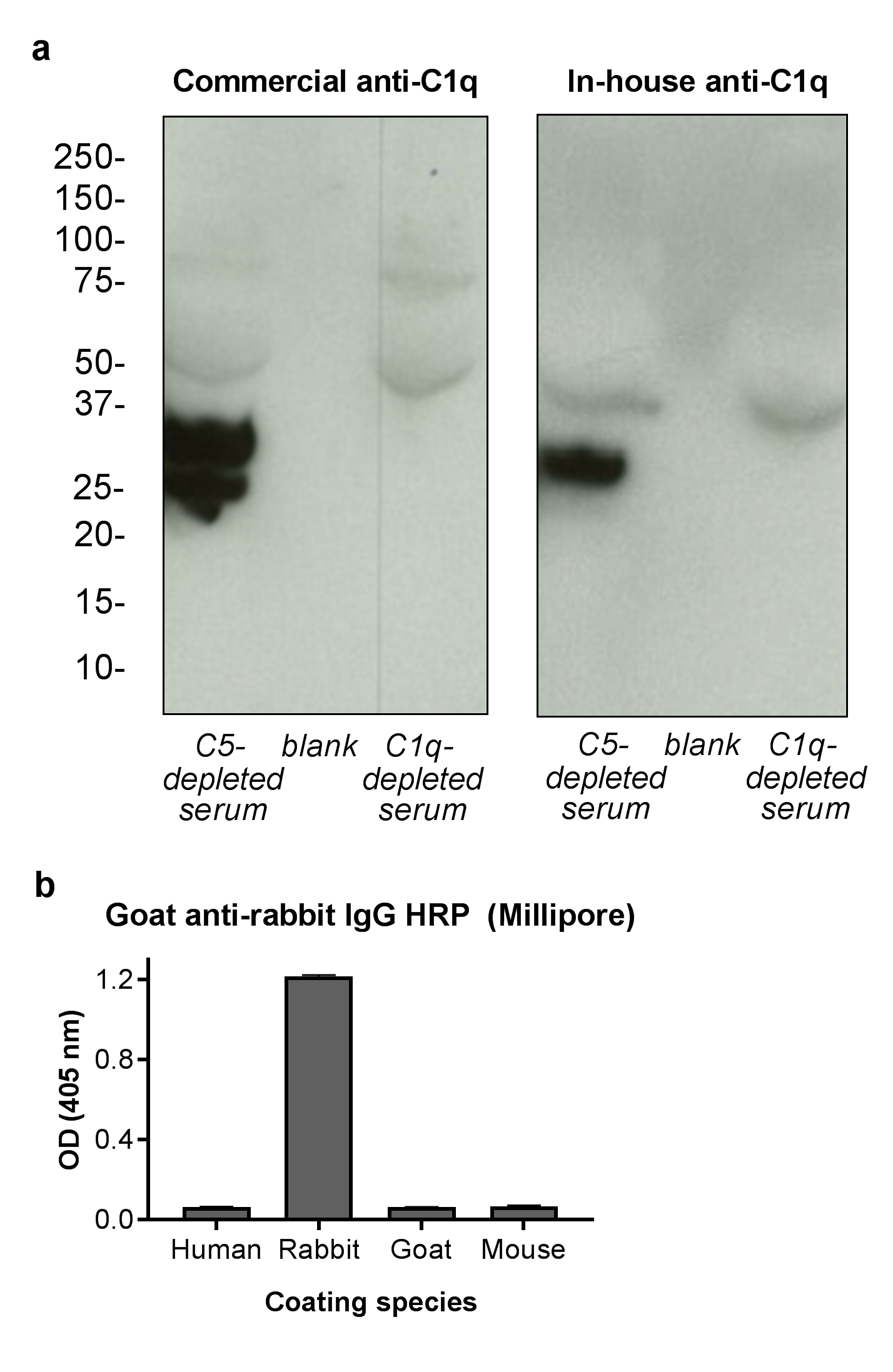


**Figure S2. Validation of detection antibodies used in C1q-fixation assay.** (**a**) Commercial and in-house rabbit anti-C1q IgG were tested for specificity and sensitivity by Western blot, and both antibodies strongly recognized C1q (A-chain 29 kDa, B-chain 27 kDa). (**b**) Commercial goat anti-rabbit IgG HRP (Millipore) was tested for specificity by standard ELISA, and was found to strongly and specifically recognize rabbit IgG only, and importantly did not cross-react with human antibodies.


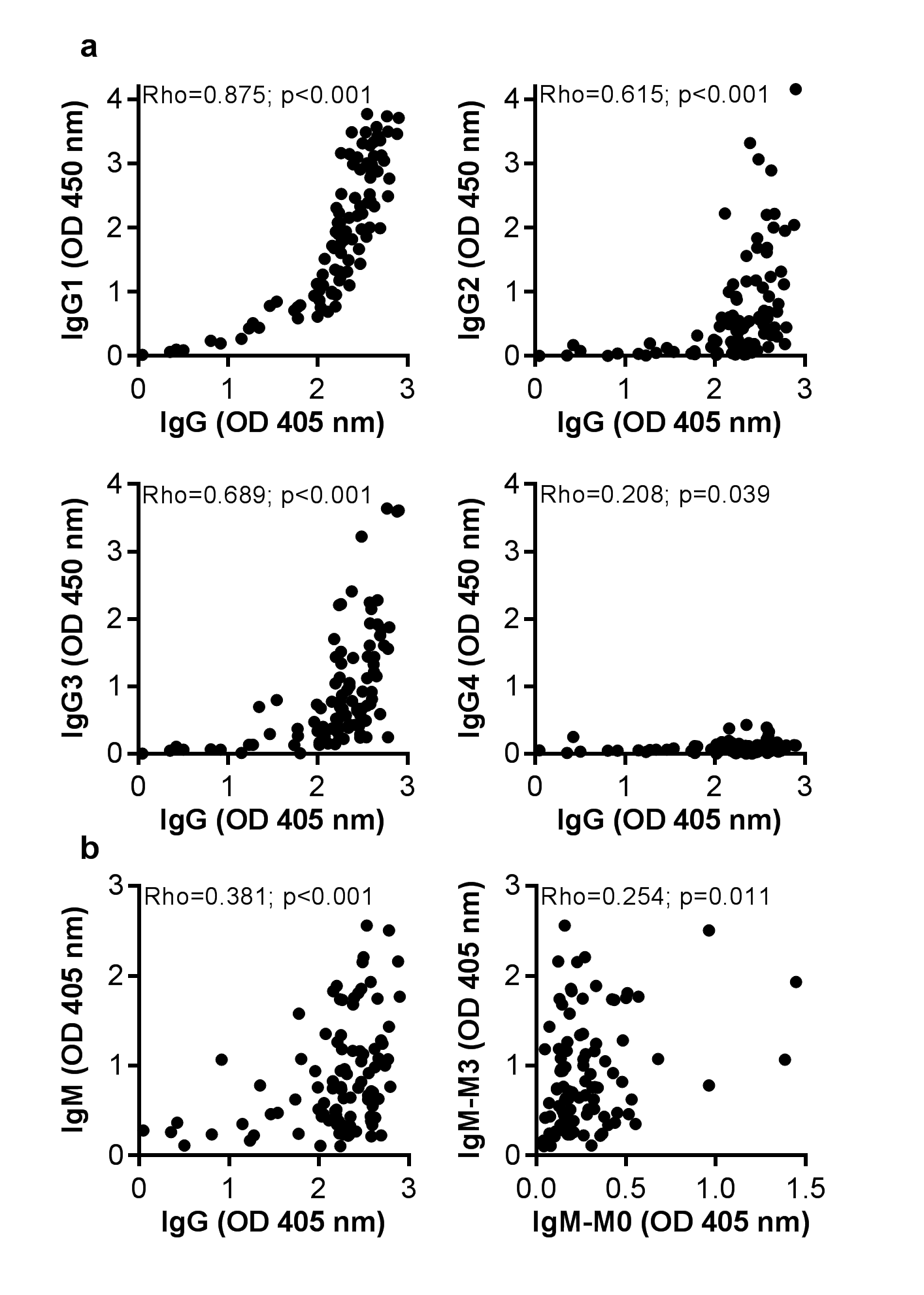


**Figure S3. RTS,S predominately induces anti-CSP IgG1, and some IgG3, IgG2 and IgM antibodies.** Children in RTS,S vaccine group from Manhiça and Ilha Josina cohorts (N=99) were tested for IgG, IgG subclasses and IgM to CSP. Sera collected after vaccination (month 3, M3) were tested and the mean of duplicates were graphed as scatter-plots, whereby (**a**) shows IgG and IgG subclasses, and (**b**) shows IgG and IgM, and IgM at M0 and M3.


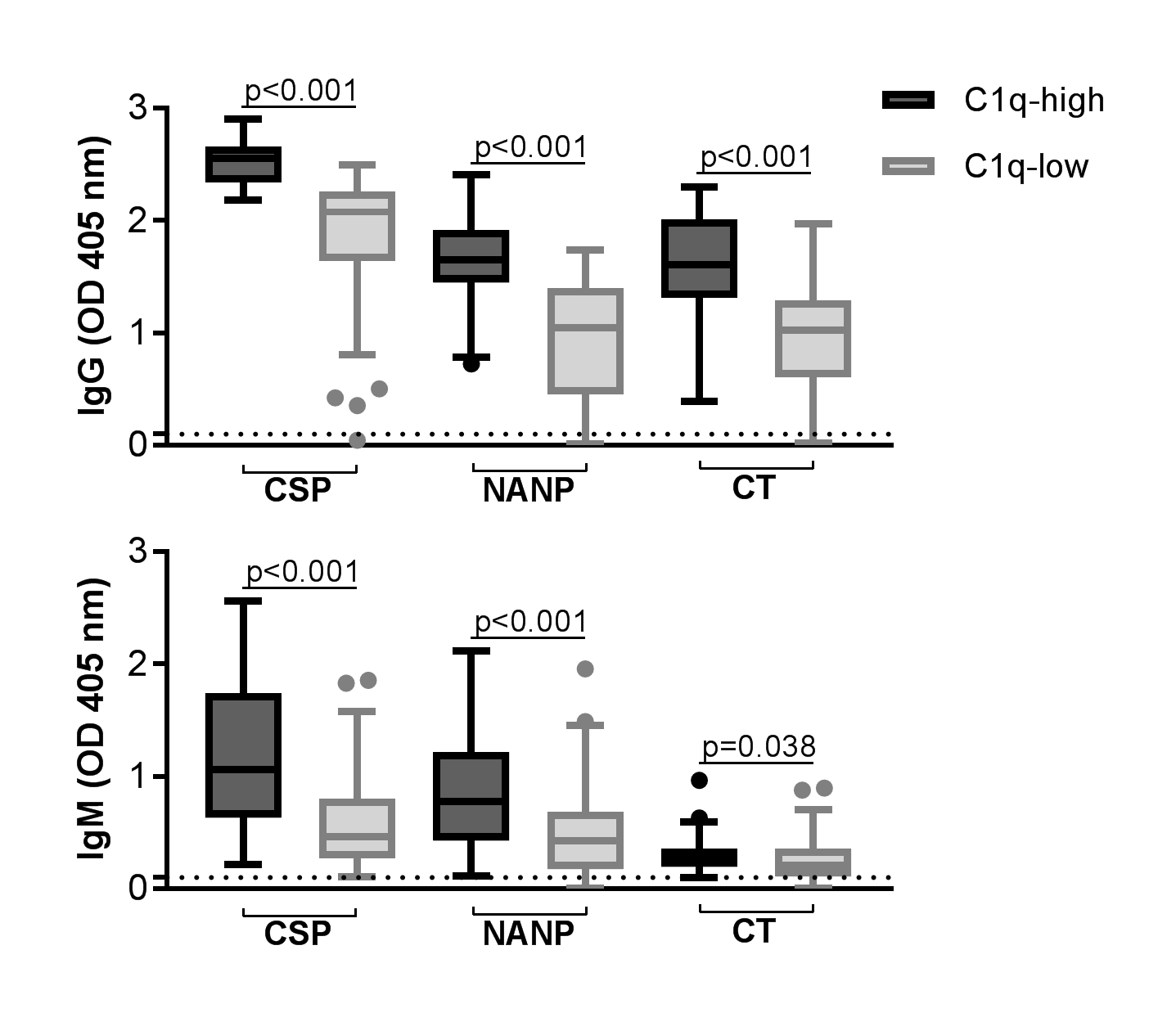


**Figure S4. Epitope-specificity of children with high or low C1q-fixing antibodies.** Children in RTS,S vaccine group from Manhiça and Ilha Josina cohorts (N=99, 3M) were tested for C1q-fixation to CSP, and categorized into high and low groups (C1q-high, OD range 2.03 to 4.00; C1q-low, OD range 0.02 to 1.97). Children in high and low groups were tested for IgG and IgM to NANP and CT. Samples were tested in duplicate, and the mean value was used to generate box-plots plots whereby top, center and bottom horizontal lines represent the 75^th^ percentile, median and 25^th^ percentile (respectively), upper and lower whiskers represent the highest and lowest values within 1.5x IQR (respectively), and values that exceed this range are presented as dots. Malaria-naïve negative controls from Melbourne donors were used to calculate the seropositivity cut-off value (dashed line). Reactivity between unpaired samples was compared using Mann-Whitney U test.

**
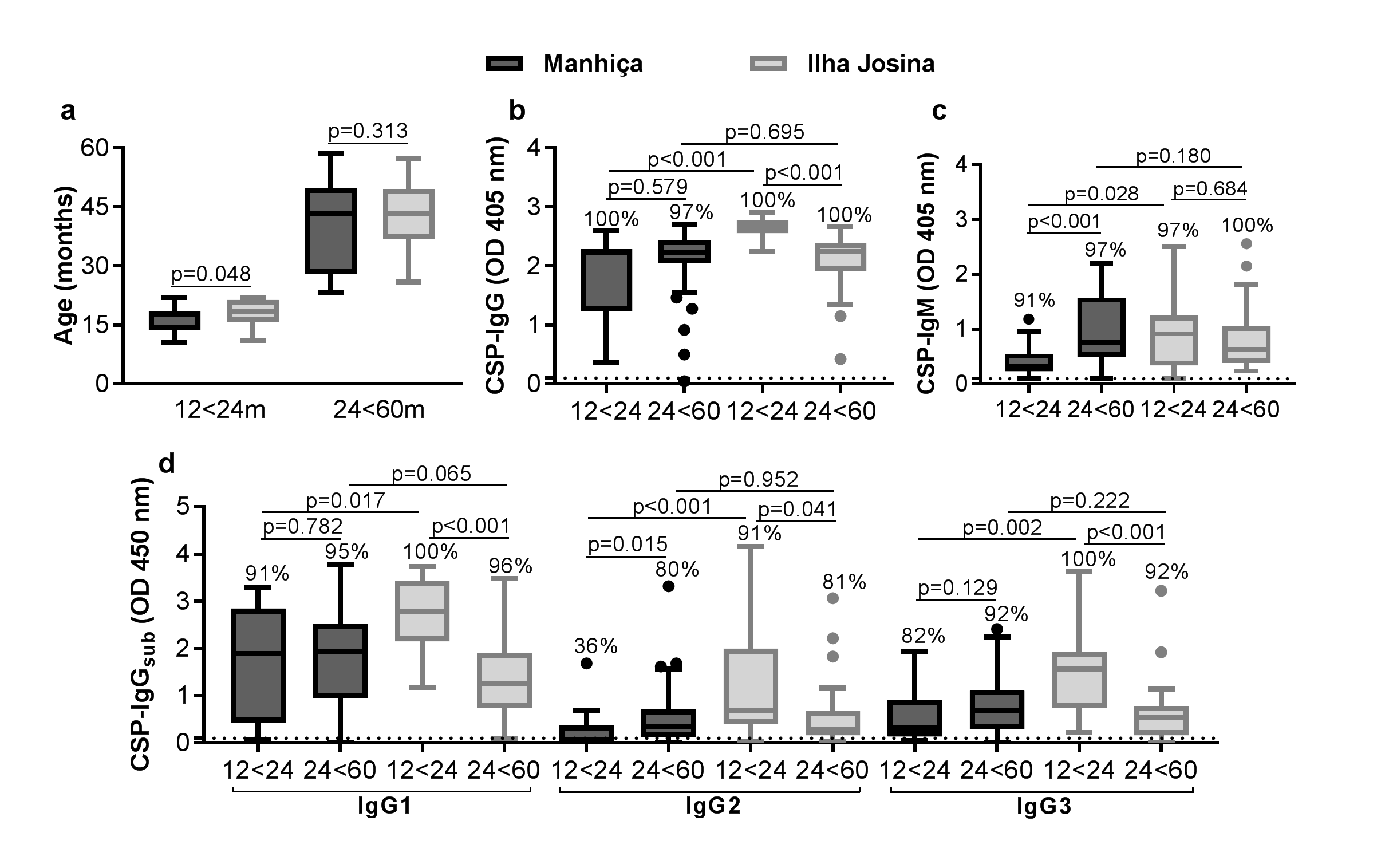

Figure S5. Younger and older children in Manhiça and Ilha Josina cohorts.** Children in RTS,S vaccine group from Manhiça (black box-plots) and Ilha Josina cohorts (grey box-plots) were categorized into younger (12 to 24 months; Manhiça n=11 and Ilha Josina n=23, respectively) and older (24 to 60 months; Manhiça n=39 and Ilha Josina n=26, respectively) age groups. (**a**) Age of children between cohorts was compared using Mann-Whitney U test. Sera collected after vaccination (month 3, M3) were tested for IgG (**b**), IgM (**c**), and IgG subclasses (**d**) to CSP. Samples were tested in duplicate, and the mean value was used to generate box-plots plots whereby top, center and bottom horizontal lines represent the 75^th^ percentile, median and 25^th^ percentile (respectively), upper and lower whiskers represent the highest and lowest values within 1.5x IQR (respectively), and values that exceed this range are presented as dots. Malaria-naïve negative controls from Melbourne donors were used to calculate the seropositivity cut-off value (dashed line), and the percentage of individuals above this threshold are shown. Reactivity between paired samples and unpaired samples were compared using Wilcoxon matched pairs signed rank test and Mann-Whitney U test, respectively.


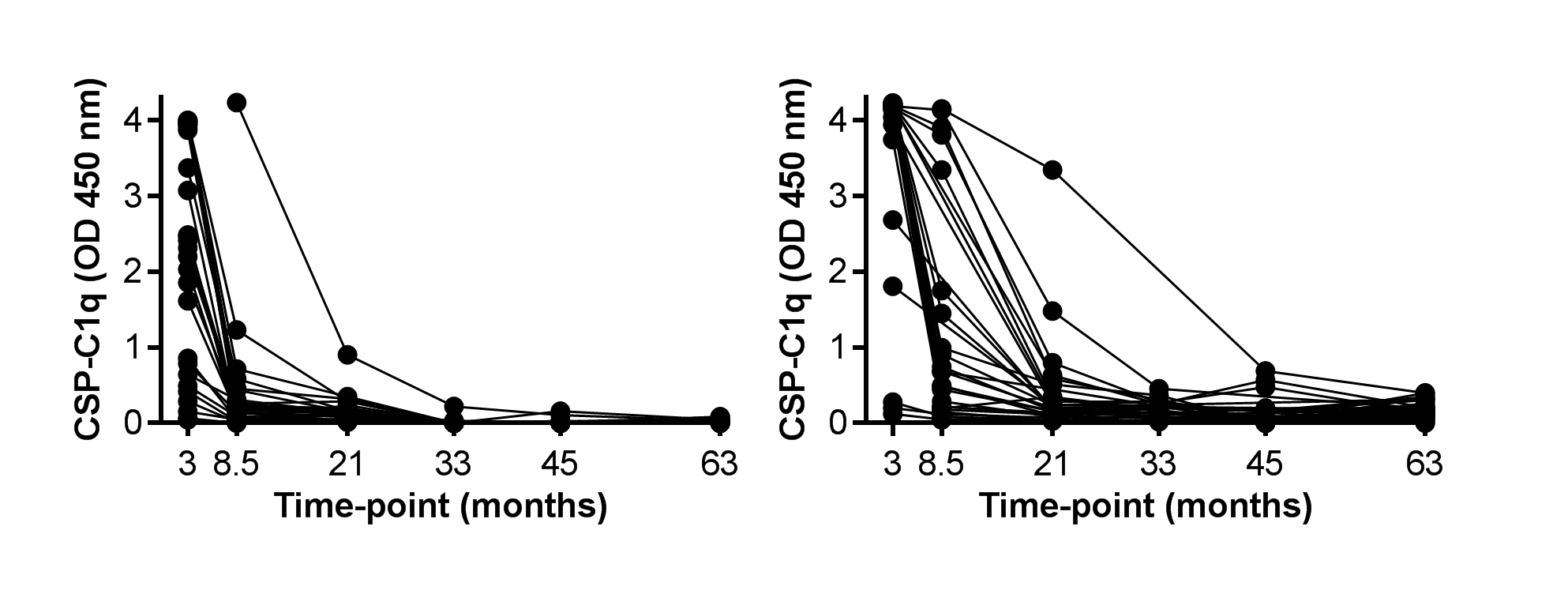


**Figure S6. Functional complement-fixing antibodies decline over time.** Random selection of children vaccinated with RTS,S (Manhiça cohort, n=30) were tested for C1q-fixation to CSP at month 3, 8.5, 21, 33, 45 and 63. Individual data points were shown for sera tested at 1/250 (left) and 1/110 (right) dilutions.


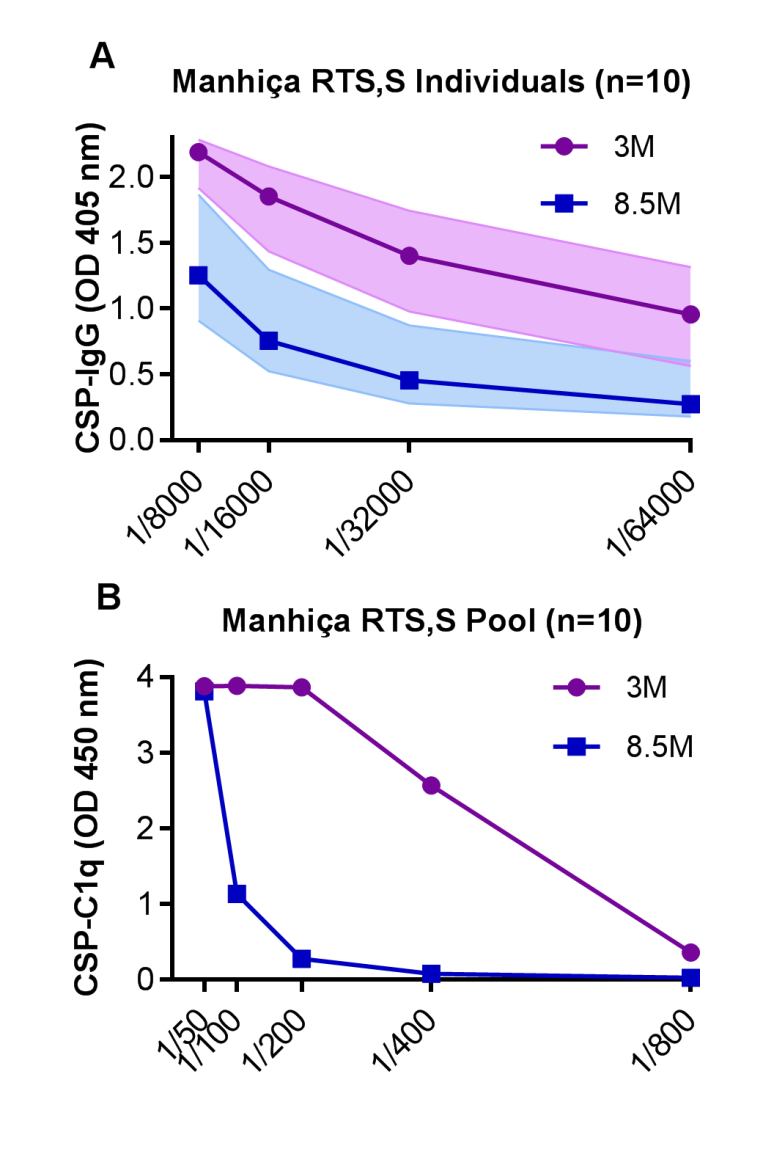


**Figure S7. Antibody concentration and functional C1q-fixation responses.** Selection of children in the RTS,S vaccine group from Manhiça cohort (n=10, M3) who demonstrated strong C1q-fixation reactivity were re-tested for IgG and C1q-fixation to CSP at month 3 and 8.5 time-points. (**a**) Individuals were tested for IgG to CSP between 1/8000 and 1/64000 dilutions in duplicate, and the median and 95% CI of the median are shown by the symbol and shaded areas, respectively. (**b**) Samples were pooled, and tested for C1q-fixation to CSP between 1/50 and 1/800 dilutions in duplicate, and the mean and range of duplicates are shown.

**Table S1. Linear regression between epitope-specific IgG and C1q-fixation to CSP, induced by vaccination with RTS,S (N=99).**

| Response | Coefficient | SE | 95% CI | p-value | Adj R-squared |
| --- | --- | --- | --- | --- | --- |
| NANP-IgG | 1.838 | 0.165 | 1.511, 2.166 | <0.001 | 0.559 |
| CT-IgG | 1.734 | 0.185 | 1.364, 2.104 | <0.001 | 0.466 |
| NANP-IgG and CT-IgG | 1.288 | 0.189 | 0.912, 1.664 | <0.001 | 0.639 |
| NANP-IgM | 1.042 | 0.272 | 0.502, 1.582 | <0.001 | 0.124 |
| CT-IgM | 1.052 | 0.794 | -0.524, 2.629 | 0.188 | 0.008 |
